# Order, please! Explicit sequence learning in hybrid search in younger and older age

**DOI:** 10.3758/s13421-021-01157-2

**Published:** 2021-04-19

**Authors:** Iris Wiegand, Erica Westenberg, Jeremy M. Wolfe

**Affiliations:** 1 Donders Institute for Brain, Behavior and Cognition, Department of Neuropsychology and Rehabilitation Psychology, Radboud University, Postbus 9104, 6500 HE Nijmegen, The Netherlands; 2 Visual Attention Lab, Brigham and Women’s Hospital, Harvard Medical School, Boston, MA USA; 3 Department of Psychology, Ludwig Maximilian University Munich, Munich, Germany; 4 Departments of Ophthalmology and Radiology, Harvard Medical School, Boston, MA USA

**Keywords:** Sequence learning, Incidental and intentional learning, Implicit and explicit memory, Visual search, Cognitive aging

## Abstract

Sequence learning effects in simple perceptual and motor tasks are largely unaffected by normal aging. However, less is known about sequence learning in more complex cognitive tasks that involve attention and memory processes and how this changes with age. In this study, we examined whether incidental and intentional sequence learning would facilitate hybrid visual and memory search in younger and older adults. Observers performed a hybrid search task, in which they memorized four or 16 target objects and searched for any of those target objects in displays with four or 16 objects. The memorized targets appeared either in a repeating sequential order or in random order. In the first experiment, observers were not told about the sequence before the experiment. Only a subset of younger adults and none of the older adults incidentally learned the sequence. The “learners” acquired explicit knowledge about the sequence and searched faster in the sequence compared to random condition. In the second experiment, observers were told about the sequence before the search task. Both younger and older adults searched faster in sequence blocks than random blocks. Older adults, however, showed this sequence-learning effect only in blocks with smaller target sets. Our findings indicate that explicit sequence knowledge can facilitate hybrid search, as it allows observers to predict the next target and restrict their visual and memory search. In older age, the sequence-learning effect is constrained by load, presumably due to age-related decline in executive functions.

**Supplementary Information:**

The online version contains supplementary material available at 10.3758/s13421-021-01157-2.

## Introduction

Thoughts and actions are often organized in sequences (Ashe et al., 2006; Lashley, 1951; Zacks & Tversky, 2001). Sequences may occur in the environment (e.g., traffic lights) or be self-created (e.g., daily morning routine). Learning such sequences allows us to anticipate events and, thus, prepare behavioral responses to perform many daily activities more efficiently (Keele et al., 2003). Sequence learning can happen incidentally through the learner’s experience. Intentional sequence learning, by contrast, involves explicit acquisition and application of retrievable knowledge about a sequential structure, as in cooking with a recipe or tying one’s tie (Meier & Cock, 2012).

Sequence learning is often investigated using a serial response-time task (SRTT). The original SRTT introduced by Nissen and Bullemer (1987) is a four-choice reaction-time  task, in which observers respond to a visual cue that appears at any one of four positions with a corresponding button press. The visual cues either appear in a repeating sequence of positions or play out in random order. After a number of repetitions, response times (RTs) typically become faster in the sequence than random conditions, which is attributed to observers starting to learn the visuo-motor pattern. The sequence in the SRTT can be learned incidentally and even implicitly (Schwarb & Schumacher, 2012). Implicit learning is inferred if a performance benefit is observed although the observer did not become aware of the sequence structure and is unable to express declarative knowledge about it (Schacter, 1987).

A number of variations of the original SRTT have been introduced. These demonstrated that the sequence-learning effect is not restricted to motor learning, but can also occur in perceptual and attentional processing stages (Mayr, 1996; Goschke, 1998; Goschke & Bolte, 2012; Remillard, 2003; Robertson & Pacual-Leone, 2001). However, this learning is not as consistent as when the motor sequence is repeated (Willingham, Nissen, & Bullemer, 1989; Nattkemper & Prinz, 1997). As compared to learning response patterns, learning of “pure” perceptual sequences takes longer and is more likely when the sequence structure is simple and participants are aware of the sequence (Abrahamse, Jimenez, Verwey, & Clerk, 2010; Deroost & Soetens, 2006a; Remillard, 2003). Furthermore, within the domain of pure perceptual learning, the learning mechanisms underlying may vary with the type of the to-be-learned perceptual features, such as target location, color, or modality (Deroost & Coomans, 2018; Deroost & Soetens, 2006b; Koch et al., 2020).

Sequence learning is further relevant for long-term memory processes. In particular, explicit sequence knowledge is used to organize episodic memory retrieval by binding temporal inter-item associations within a series of to-be-remembered items (Kahana, 1996). It involves the establishment and retrieval of temporal-order memory representations, which are considered a type of context memory that relies on executive control functions (Shimamura, 1995). Such explicit sequence learning based on item-item associations in memory tasks has been shown to decline with age (Allen et al., 2015; Cabeza et al., 2000). This supports the assumption that the cognitive and neural mechanisms underlying explicit memory processes are particularly affected by aging (Craik & Jennings, 1992; Mitchell, 1989; Nilsson, 2003). By contrast, performance in memory tasks that rely on declarative knowledge and executive control to a lesser extent are often spared in older age (Prull et al., 2000; Schugens et al., 1997). Accordingly, several studies have demonstrated that incidental sequence learning in SRTTs is preserved in older age (Cherry & Stadler, 1995; Dennis, Howard, & Howard, 2006; Howard & Howard, 1989, 1997; Negash et al., 2003), at least for simple visuo-motor and visuo-spatial sequences (Howard et al., 2004). However, in these incidental learning tasks, older adults were not as likely as younger adults to acquire explicit awareness of the sequence, supporting the idea that there is an age-related decline in declarative aspects of learning and memory of temporal orders (Dennis et al., 2006; Howard & Howard, 1989, 1997). In visual search, implicit memory for spatial configurations (“contextual cueing”; Chun & Jiang, 1998) improves performance in older as well as younger adults (Howard et al., 2004). Additionally, younger and older adults show similar repetition priming effects. When the target features or locations repeat over consecutive trials, RTs are faster than when features or locations change (Madden et al., 2005; McCarley et al., 2004; Wiegand et al., 2013). Whether the sequential order of target features or locations influences visual search in older adults, however, has not been investigated yet.

## The present study

Previous research has demonstrated effects of sequence learning on multiple cognitive processes and suggests that those effects vary with age. A common interpretation of sequence-learning effects is that behavioral adaptation to environmental regularities helps with the execution of complex behavior in the real world. However, prior studies have focused on sequence-learning effects on isolated cognitive processing components and typically used simple, abstract stimulus material. In the present study, we tested sequence-learning effects, and potential age differences therein, in a single complex search task with realistic stimulus material. Specifically, we incorporated sequence learning into a “hybrid search” task (Schneider & Shiffrin, 1977), which is a combination of visual and memory search. The task draws on both selective attention and long-term memory processes and is more akin to many of the searches we perform in the real world, for example, holding a shopping list in memory as we search the shelves of a store (Boettcher et al., 2018; Wolfe et al., 2016). In the hybrid search task re-introduced by Wolfe (2012), observers first memorize a number of target objects. These are real-world photographs that are easy to commit to long-term memory (Konkle et al., 2010; Standing et al., 1970). Then, observers search visual displays for an instance of any of those targets among distractor objects. Wolfe (2012) found that RTs in hybrid search increased linearly with the number of distractors in the visual display (i.e., visual set size) and increased logarithmically with the number of target objects held in long-term memory (i.e., memory set size). The slopes of these RT × set size functions provide a means to measure efficiency of visual search and memory search in one task, with steeper slopes indicating less efficient processing.

We recently examined age differences in hybrid search. While there was evidence of general age-related slowing, we demonstrated similar RT × set size functions for younger and older adults: The relative costs of adding distractors to the display and adding targets to the memory set were similar for both age groups (Wiegand & Wolfe, 2020), even up to high set sizes (64 items; Wiegand et al., 2019). This suggests no qualitative age differences in processing in the standard task version, where targets from the memory set appear in random order across trials. Thus, the task is well suited to be used to test for age differences in sequence-learning effects on attention and memory processes, as we know that the baseline performance level is comparable between younger and older adults in hybrid search.

**Fig. 1:**
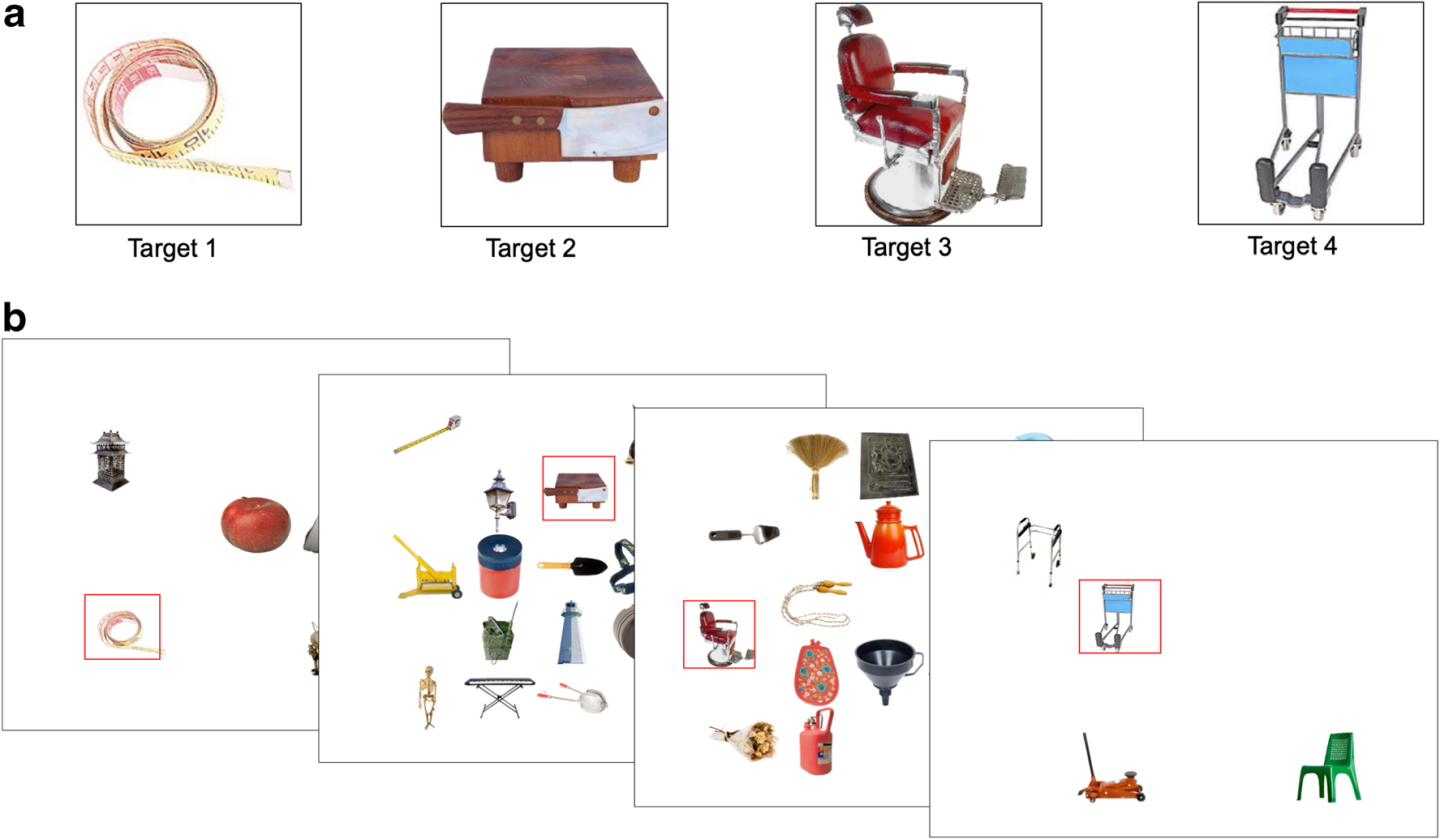
**A** Example of a four-target memory set. The target objects were first memorized during a learning phase after which recognition memory for them was tested at least twice. **B** Four search trials. Targets are highlighted in red which, of course, would not be the case in the actual experiment. In the search tasks, participants located one target object on each trial and used the mouse to click on it as quickly as possible

To examine sequence learning in hybrid search, we used a target localization version of the task. In the localization task, a target is present on every search trial and selected by the observer via mouse click. Observers memorized either four or 16 target objects (“memory set size”) and then searched for any of those targets in displays composed of four or 16 objects (“visual set size”; see Fig. 1). The targets either followed a sequence that repeated over trials or occurred in a random order over trials within a block. We measured RT under incidental learning conditions (Experiment 1) and intentional learning conditions (Experiment 2) and assessed observers’ explicit knowledge of the target sequence after the search task. We hypothesized that learning the sequence of targets would facilitate search by enabling the observer to anticipate the next upcoming target. This would allow observers to restrict memory search and to guide visual search for this target. Increased efficiency in visual and memory search by sequence learning would manifest in faster RTs in the sequence compared to random blocks. We further examined whether the sequence-learning effect in hybrid search would change with age. Similar learning effects in younger and older adults could be expected if sequence learning happened incidentally and relatively effortless. However, if sequence learning is supported by explicit knowledge about the target sequence, one could expect an age-related reduction in the sequence effect due to impaired acquisition of explicit knowledge and executive control of memory retrieval in older compared to younger adults. The latter might further be affected by the length of the sequence. An explicit representation of the four-target sequence might be acquired more easily than of the 16-target sequence, and this load effect might be more pronounced in older than younger age.

## Methods

### Participants

For Experiment 1, we collected data from 25 younger participants between the ages of 18 and 35 years and 13 older participants between the ages of 65 and 85 years. More younger than older participants were recruited because we analyzed within-group differences (between learners and non-learners) in sequence learning in the younger sample only (see *Results* section below). For Experiment 2, we collected new data from 14 younger participants between the ages of 18 and 35 years and 13 older participants between the ages of 65 and 85 years. Our sample size was based on prior investigations using hybrid search tasks to compare younger and older adults (Wiegand & Wolfe, 2020). We further conducted an a priori power analysis using G*Power (Faul et al., 2007) to test for a within-subject and a within-between interaction effect in a mixed ANOVA with eight (2 × 2 × 2) correlating repeated measures (r = .50). The correlation between measures was based on prior studies (Wiegand & Wolfe, 2020). This analysis revealed that a total sample of 16 participants was required to achieve a power of .80, a medium effect size (η^2^=.06), and an alpha of .05. Although we did not conduct a priori power analyses for analyses of within-group comparisons in Experiment 1, subsequent compromise power analyses using G*Power show that we had power of .60 to observe these.

The participants were recruited through clinical trial recruitment announcements from Partners Healthcare and Brigham and Women’s Hospital, Harvard University (only younger adults) and advertising in the magazine FiftyPlus Advocate (only older adults). Data were collected in accordance with the Declaration of Helsinki ethical principles. Participants took part voluntarily, gave their informed consent, and were paid $11 per hour for their time. The Partners Healthcare Corporation Institutional Review Board approved all experimental procedures. We recruited different participants for Experiments 1 and 2, so none of the observers participated in both studies.

All participants had 20/25 or better corrected vision, as assessed with the ETDRS Near Vision Chart (Bailey & Lovie, 1976), and none were colorblind, as assessed by the Ishihara Test (Ishihara, 1980). Participants were excluded if they reported having been diagnosed with any neurological, psychiatric, or chronic somatic disorder. In Experiment 1, one older participant was excluded based on this criterion. In Experiment 2, two younger participants were excluded based on this criterion. All participants were further screened for the presence of mild to severe depressive symptoms using the Center for Epidemiologic Studies Depression Scale (CES-D; Radloff, 1977), and older participants were additionally screened for symptoms of dementia onset using the Mini-Mental State Examination (MMSE; Folstein et al., 1975). No participant had a CES-D score higher than 20, indicating no symptoms of moderate or severe depression. In Experiment 2, all older participants except one scored higher than 26 in the MMSE, indicating that they showed no early signs of dementia. This older participant had a score of 24 and was therefore excluded. In Experiment 1, one younger participant was further excluded for not completing the experiment. In Experiment 2, one older participant was excluded for not completing the experiment. The final participant samples for analysis therefore consisted of 24 younger adults and 12 older adults in Experiment 1 and of 12 younger adults and 11 older adults in Experiment 2.

Once participants were screened for inclusion criteria, we further assessed demographic information (age, sex, education) with a questionnaire and measured cognitive and visuo-motor speed with the Digit-Symbol Substitution Test (DSST; Wechsler, 1958). For native English speakers (17 younger and 11 older adults in Experiment 1, nine younger adults and 11 older adults in Experiment 2), verbal abilities (verbal IQ) were measured with the North American Adult Reading Test (NAART; Blair & Spreen, 1989; Nelson, 1982). Additionally, we assessed all participants’ subjective cognitive failures in everyday tasks with the Cognitive Failures Questionnaire (CFQ, Broadbent et al., 1982) and the older participants’ cognitive reserve^1^ with the Cognitive Reserve Index questionnaire (CRIq; Nucci et al., 2012). A comparison of the demographic information of all participants from Experiment 1 and Experiment 2, including the screening tests, can be found in Tables 1 and 2, respectively.

**Table 1:** Demographic information and questionnaire scores for the younger and older participants who took part in Experiment 1, and statistical group comparisons

Participant characteristics Experiment 1			
	Younger adults (n=24)	Older adults (n=12)	
Age (years)	23.71 (3.92)	71.92 ± 6.10	t=28.05, p<.001
Gender	18 female, 6 male	8 female, 4 male	*χ*^2^=0.28, p=.60
Handedness	22 right, 1 left, 1 both	12 right	*χ*^2^=1.64, p=.65
CES-D	6.50 (6.30)	5.92 (6.08)	t=0.26, p=.79
MMSE	Not acquired	28.75 (1.48)	--
DSST*	69.26 (9.94)	45.58 (10.47)	t=6.57, p<.001
CFQ	29.46 (11.85)	21.67 (11.42)	t=1.88, p=.07
NAART (VIQ)**	110.56 (6.05)	120.10 (6.15)	t=4.33, p<.001
CRIq	Not acquired	132.58 (16.62)	--

All values, excluding gender and handedness, indicate the mean and standard deviation (in parentheses) of the samples

* The young adult learners had higher DSST scores compared to young adult non-learners (Learners: 76.20 (7.43); Non-learners: 63.92 (8.29)). The subgroups did not differ significantly in any of the other variables

**NAART scores were only acquired for native English speakers (17 younger adults and 11 older adults)

*CES-D* Center for Epidemiologic Studies Depression Scale, *CFQ* Cognitive Failures Questionnaire, *CRIq* Cognitive Reserve Index questionnaire, *DSST* Digit Symbol Substitution Test, *MMSE* Mini-Mental State Examination, *NAART (VIQ)* North American Adult Reading Test (Verbal Intelligence Quotient)

**Table 2:** Demographic information and questionnaire scores for the younger and older participants, who took part in Experiment 2, and statistical group comparisons

Participant characteristics Experiment 2			
	Younger adults (n=12)	Older adults (n=11)	
Age (years)	26.50 (2.97)	67.72 (3.29)	t=31.60, p<.001
Gender	10 female, 2 male	6 female, 5 male	*χ*^2^=2.25, p=.13
Handedness	12 right	9 right, 1 left, 1 both	*χ*^2^=2.39, p=.30
CES-D	5.67 (3.68)	5.27 (4.12)	t=0.24, p=.81
MMSE	Not acquired	29.09 (1.30)	--
DSST	71.00 (8.73)	53.00 (9.30)	t=4.79, p<.001
CFQ	22.92 (8.84)	26.54 (10.01)	t=0.92, p=.37
NAART (VIQ)*	112.98 (7.15)	116.51 (4.09)	t=2.03, p=.06
CRIq	Not acquired	142.09 (13.00)	--

All values, excluding gender and handedness, indicate the mean and standard deviation (in parentheses) of the samples

*NAART scores were only acquired for native English speakers (nine younger adults and 11 older adults)

*CES-D* Center for Epidemiologic Studies Depression Scale, *CFQ* Cognitive Failures Questionnaire, *CRIq* Cognitive Reserve Index questionnaire, *DSST* Digit Symbol Substitution Test, *MMSE* Mini-Mental State Examination, *NAART (VIQ)* North American Adult Reading Test (Verbal Intelligence Quotient)

### Apparatus and stimuli

We retrieved our target and distractor objects from a database of distinct real-world objects that was compiled by Brady et al. (2008). The database contains 2,400 unique objects. We decided to exclude some of these images according to the following criteria: (1) white or translucent objects that were not sufficiently distinct from the background, (2) images including words, numbers, or arrows, (3) images with part of the object cut off, (4) objects that were too similar to other objects, (5) images containing pictures of landscapes or humans, or (6) objects that evoked a strong feeling of disgust or dislike, as reviewed by other experimenters in the lab. We excluded 479 objects according to the criteria listed above, leaving 1,921 objects in total. The experiment was run on an iMac monitor (model A1225; EMC 2211) with a 24-in. screen. The computer was running OSX Version 10.11.6. The experiment was written with and run in MATLAB 7.10.0 and 9.0 using Psychtoolbox version 3.0.9 (Brainard, 1997).

### Experimental procedure

Our experiments were a variant of the “hybrid” visual and memory search task introduced by Wolfe (2012), in which participants localized one of several target objects among distractor objects (see Wolfe, 2012, Experiment 3). As noted earlier, Wolfe (2012) showed that RTs in hybrid search tasks increase linearly with the visual set size and increase logarithmically with the memory set size. This held for target localization tasks as well as for target present/absent tasks. The two types of task produce comparable RT data. In the present sequence learning hybrid search task, we used the localization task because it involves a target on every trial. We thought that the presence of target-absent trials in the present/absent task would hinder the learning of target sequences. Previous studies using versions of the SRTT have shown that learning is more difficult when unpredictable events are interspersed in the repeating sequence, especially in older observers (Howard et al., 2004; Howard & Howard, 1997)

Our version of the hybrid search task was divided into four blocks: two with random presentation of targets and two with sequential presentation of targets. Within each condition, one block had a memory set size of four targets and one block had a memory set size of 16 targets. The positions of the targets and distractors varied randomly. The order of blocks was pseudo-randomized for each participant to control for order effects.

Each experimental block began with the serial presentation of four or 16 targets, which the participants were instructed to memorize (Fig. 1). A memory test then followed, with targets comprising 50% of the images and non-targets comprising the rest. Participants were instructed to respond with button presses as to whether or not the image displayed was one of the targets they had memorized. Participants received feedback about their correctness after each response. They had to respond with at least 90% accuracy, twice in a row, in order to successfully complete the memory test. Thus, the minimum number of memory tests was two. If participants failed this criterion in the second test, they were again presented with the targets and completed another memory test. On average, younger participants completed 2.25 tests with an average accuracy of 99% in the final test. Older participants completed 2.18 tests on average with an average accuracy of 99% in the final test. Thus, both groups were easily able to encode the memory sets.

After completion of the memory test, participants advanced to the experimental search task. Participants were instructed to search the screen to find one of the objects they had memorized among other distractor objects, and to click on the target as quickly as possible with their computer mouse (Fig. 1). Trials contained a visual set size of either four or 16. Thus, each trial contained one target and three or 15 distractor objects. Distractors were drawn randomly from a large set of object images and did not repeat over trials. Equal numbers of trials at each visual set size were presented in random order within a block having a single memory set size. If the wrong target or area of the screen was clicked, a high-pitched beep indicated that the participants had made an error, and if they clicked correctly, a lower-pitched beep was played before the display advanced to the next trial. Each search block started with ten practice trials to familiarize the participants with the task and to reassure that they were capable of controlling the mouse. The number of experimental search trials was chosen such that each sequence was repeated 20 times. Thus, blocks with a memory set size of four contained 80 search trials and blocks with a memory set size of 16 contained 320 search trials. For the random blocks, the same number of trials was used. In the random condition, targets appeared in random order during the learning and recognition stages as well as the search trials. For the fixed sequence condition, the order in which the targets appeared during the trials was the same during search as in the learning and recognition stages and the sequence was repeated 20 times.

The procedures for Experiments 1 and 2 were identical except for an important difference in the instructions. In Experiment 1, participants were not informed about the possibility that targets could appear in a repeating sequence. In Experiment 2, before each of the four blocks, participants were told whether the targets in the upcoming block would be presented in sequential or random order. They were not told the actual sequence.

In both Experiment 1 and Experiment 2, participants performed a two-alternative forced-choice (2AFC) test after the search task, to test for their acquired knowledge of the sequence. They were shown an image from one of the memorized target sequences, and then were presented with two other images from the sequence, one of which correctly followed the initial image. Each participant completed eight trials with randomly selected targets from the two sequence conditions, four for each memory set size. In Experiment 1 participants filled out an additional questionnaire about their experiences to find out if they noticed the sequence (see Online Supplementary Material I) before they began the 2AFC test. This was unnecessary in Experiment 2, as participants already knew about the sequence.

### Statistical analyses

For the search task, we analyzed outlier-corrected RT and z-transformed RT (zRT) data. Trials with RTs larger than 2.5 standard deviations from the mean were excluded. This was less than 1% of the data. Trials in which the first click was not on the target were excluded. This was less that 3% of the data. The z-transformation controlled for individual, and, thus, age differences in baseline RT (Faust et al., 1999). In this analysis, within each individual, the overall mean was subtracted from each condition’s mean, and divided by the standard deviation of the condition’s mean. Each individual’s condition z-scores greater than zero represent slower responses, whereas z-scores lower than zero represent faster responses, relative to this individual’s mean. The resulting standardized values allowed us to compare the relative condition differences between individuals independent of individual differences in mean raw RT, including overall age-related slowing.

All statistical analyses were computed in JASP (http://www.jasp-stats.org). We ran mixed ANOVAs with the factors Target Order (sequence, random), Age (younger, older), Visual Set Size (4, 16), and Memory Set Size (4, 16). Initially, we included the number of repetitions of the sequence as another factor in the ANOVA to examine whether the learning effect evolves over time. This was not the case; therefore, we discarded this factor for the sake of brevity. The repetition effects are reported in the Online Supplementary Material II. Interactions were followed up with ANOVAs and t-tests. For the latter, Bonferroni correction for multiple comparisons was applied.

We analyzed performance in the 2AFC to assess observers’ explicit knowledge about the sequence, using a one-sample t-test to test whether performance was significantly different from chance level (50%). Then, we compared performance between younger and older adults and between different sequence lengths (memory set sizes of four and 16) with mixed ANOVA with the factors Age and Memory Set Size.

For all analyses, we also calculated the Bayes factor (BF) as an estimate of how strongly the data support not only the presence of a hypothesized effect, but also how strongly a null effect is supported. BF_01_ was computed as evidence for H0/H1 and BF_10_ as evidence for H1/H0 (i.e., 1/BF_10_). Thus, BF_01_>1 indicates support for H0 (null model) and BF_10_>1 indicates support for the H1. The H1 assumed an effect of Target Order. We interpret BF according to Kass and Raftery (1995). BFs of 1–3 indicate only scarce support for a hypothesis. BFs of 3-20 indicate considerable evidence. BFs between 20 and 150 indicate strong evidence, and BFs >150 indicate very strong evidence for a hypothesis. The number of possible models in mixed designs such as ours is huge. According to the recommendations of Rouder et al. (2012, 2017) and Wagenmakers et al. (2018), in the analyses of the search performance data, we therefore specifically tested for the main effects and interactions involving Target Order and Age. The factors Visual Set Size, Memory Set Size, and their interactions, were always included in the null model.

## Results and discussion

### Experiment 1: Incidental learning

In the first experiment, participants were unaware of the target sequence before the experiment. This allowed us to test (1) whether learning would occur incidentally, indicated by faster RTs in sequence compared to random blocks, and (2) whether explicit knowledge of the sequence could be acquired, indicated by the post-experimental tests.

**Fig. 2:**
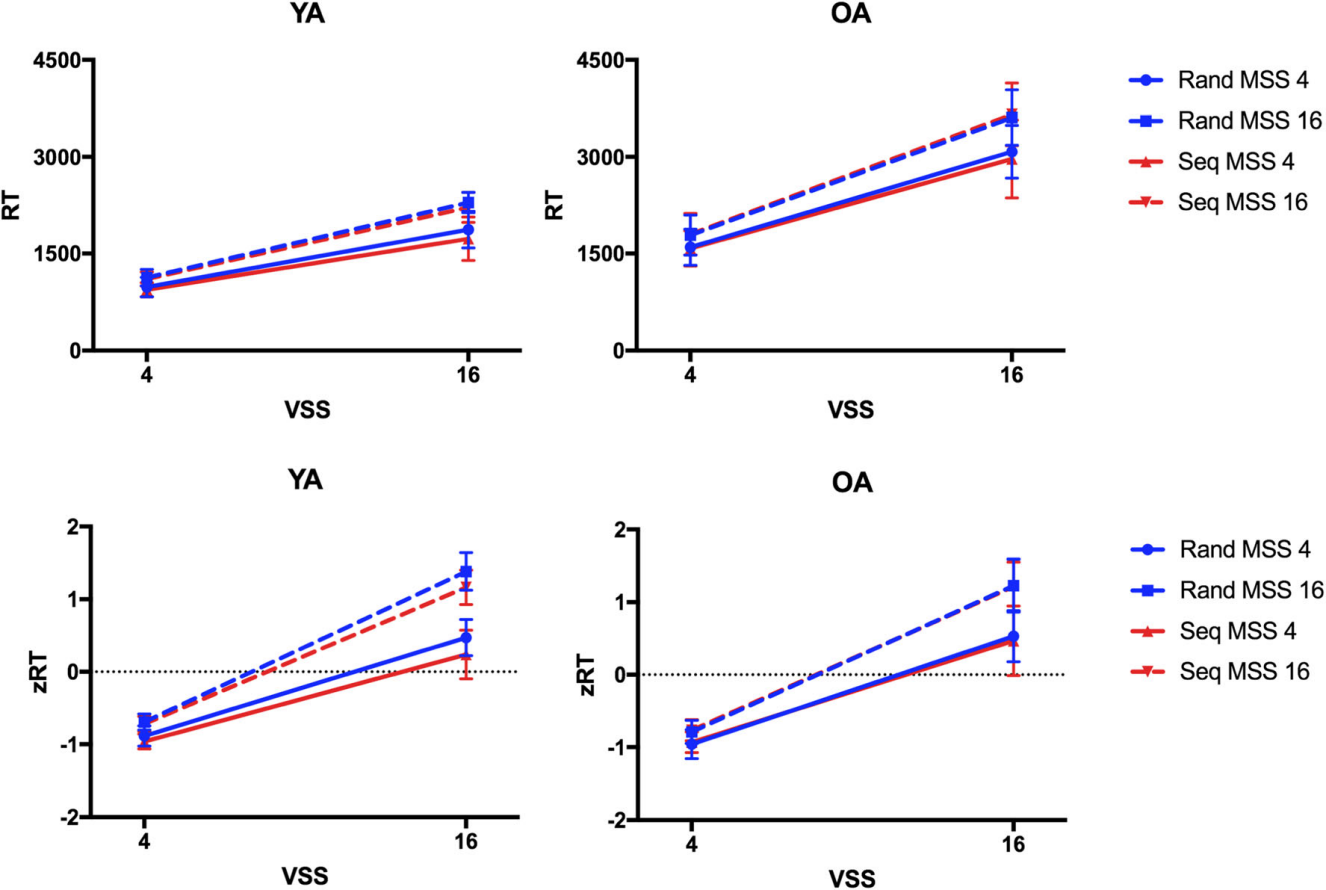
Reaction times (RTs) and z-transformed RTs (zRTs) in Experiment 1. Mean RTs and zRTs are plotted for younger adults (YA) and older adults (OA) as a function of visual set size (VSS) comparing the blocks in which targets appeared in a repeating sequence (Seq, red lines) and in which targets appeared randomly (Rand, blue lines) across trials for smaller and larger memory set sizes (MSS). Error bars indicate standard error of the mean

Figure 2 shows that younger adults responded slightly faster in the sequence blocks than in the random blocks, while no RT difference between blocks was visible in the older adults. However, the four-way ANOVA on RT across all observers did not reveal significant effects of Target Order nor interactions between Target Order and Age (all F(1,34)<1.28, p>.25, η_p_^2^<.04, BF_01_>2.84). As expected, the ANOVA revealed main effects and interactions of Visual Set Size and Memory Set Size (all F>54.87, p<.001, η_p_^2^>.61, BF_10_>275.25), such that as memory or visual set size increased, RT increased. Furthermore, the main effect of Age (F(1,34)=122.18, p<.001, η_p_^2^=.78, BF_10_=4.64e+7) and the Visual Set Size × Age interaction (F(1,34)=78.88, p<.001, η_p_^2^=.70, BF_10_=6.39e+102) were significant, indicating slower RTs and steeper search slopes in older than younger adults. The same ANOVA on zRT also did not reveal significant effects of Target Order nor interactions between Target Order and Age (all F(1,34)<1.46, p>.20, η_p_^2^<.02). Only the BF indicated some evidence for the effect of Target Order (BF_10_=3.73), but not for any further interactions including the factor (all BF_01_>1.20). The main effects and interactions of Visual Set Size and Memory Set Size (all F>49.31, all p<.001, all η_p_^2^>.59, BF_10_=2.26e+106) were significant, but there was no main effect of Age (F(1,34)=0.05, p=.83, η_p_^2^<.001; BF_01_=6.09), and the Age × Visual Set Size interaction only approached significance (F(1,34)=4.11, p=.05, η_p_^2^<.10; BF_01_=18.90).

**Fig. 3:**
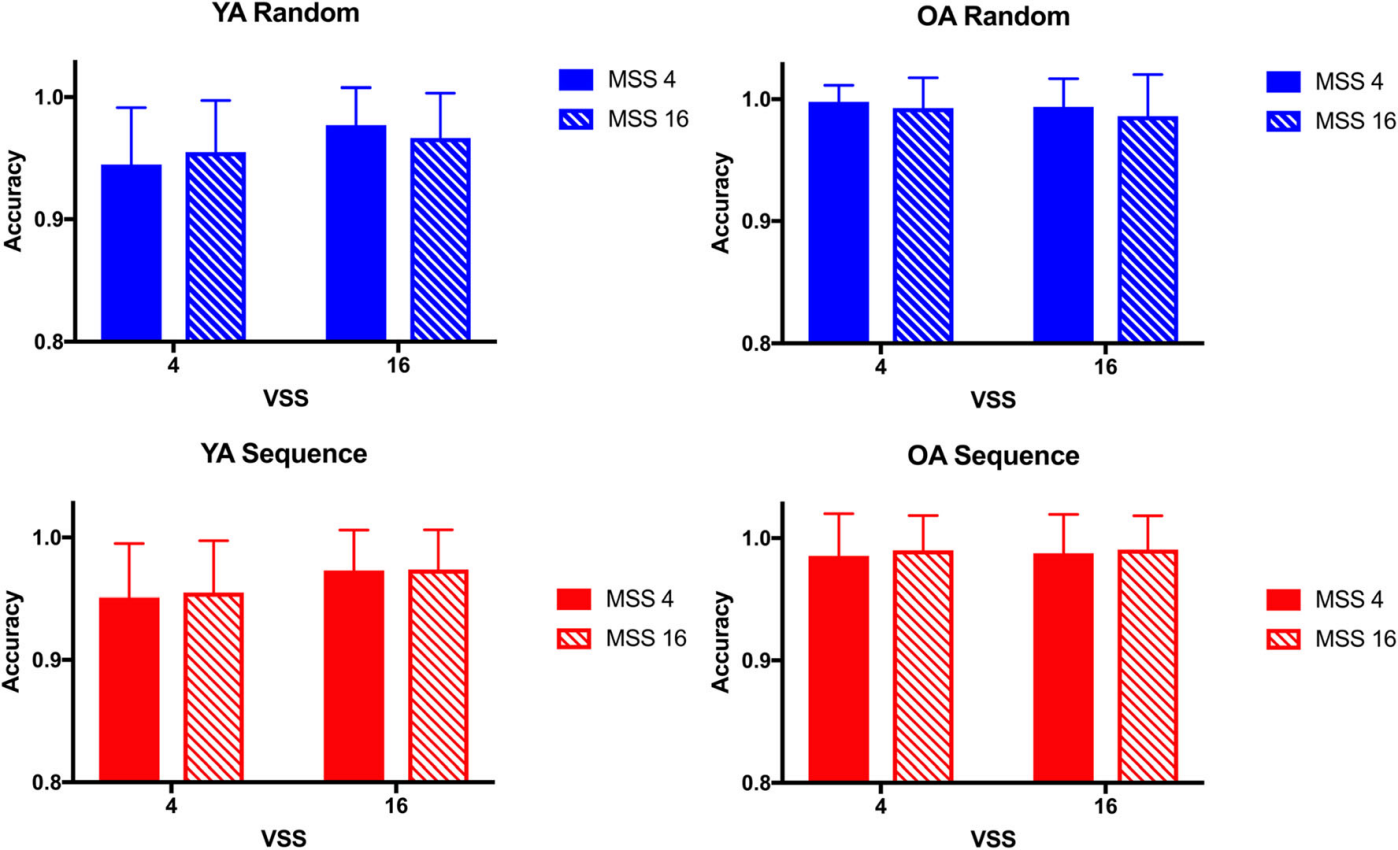
Accuracy (rate of correct clicks) in Experiment 1. Mean accuracy is plotted for younger adults (YA, left) and older adults (OA, right), comparing the blocks in which targets appeared in a repeating sequence (red bars, lower panels) and in which targets appeared randomly (blue bars, upper panels), for smaller and larger visual set sizes (VSS) and memory set sizes (MSS). Error bars indicate standard error of the mean

RT is the main variable of interest in the hybrid search task. In the localization task, error rates are very low (see also Wolfe, 2012) and varied between 0.1% and 5.5% across age groups and experimental conditions in the present experiment. Figure 3 shows the accuracy data for the different age groups and experimental conditions. We used an arcsine transformation on the accuracy data (proportion of correct trials in which the first click was on the target) to achieve greater homogeneity of the variances in the proportional data (Hogg & Craig, 1995). The ANOVA on transformed accuracy data revealed no main effect or interaction involving Target Order (all F(1,34)<3.30, all p>.07, all η_p_^2^<.03, all BF_01_>5.72), suggesting no sequence-learning effects on errors in the hybrid search task. There were significant main effects of Visual Set Size, Memory Set Size, and Age, and a significant Age × Visual Set Size interaction (all F(1,34)>8.85, all p<.006, all η_p_^2^>.20, all BF_10_>6.76). Younger adults made more errors that older adults (3.8% vs. 1.0%), suggesting a modest speed-accuracy trade-off. Across age groups, performance was slightly better in the conditions with larger memory set sizes and smaller visual set sizes. Younger adults made significantly more errors in trials with a visual set size of four than in larger displays with 16 items (t(23)=6.29, p<.001, *d*=1.2), but the Bayes factor did not support evidence for this effect (BF_10_=0.86). No other main effects or interactions were significant.

The average performance in the explicit sequence knowledge test was 53% and 52% for younger and older adults, respectively. Performance was not different from chance level of 50% (t(35)=0.50, p=.62, *d*=0.08, BF_01_=4.97). The ANOVA revealed insignificant effects of Age (F(1,34)=0.003, p=.95, η_p_^2^<.001, BF_01_=2.74), Memory Set Size (F(1,34)=1.02, p=0.32, η_p_^2^=.008, BF_01_=2.64), and the interaction of both factors (F(1,34)=0.008, p=0.93, η_p_^2^<.001, BF_01_=3.02). Thus, neither younger nor older adults demonstrated explicit sequence knowledge in the 2AFC test, neither for shorter nor longer sequences.

**Fig. 4:**
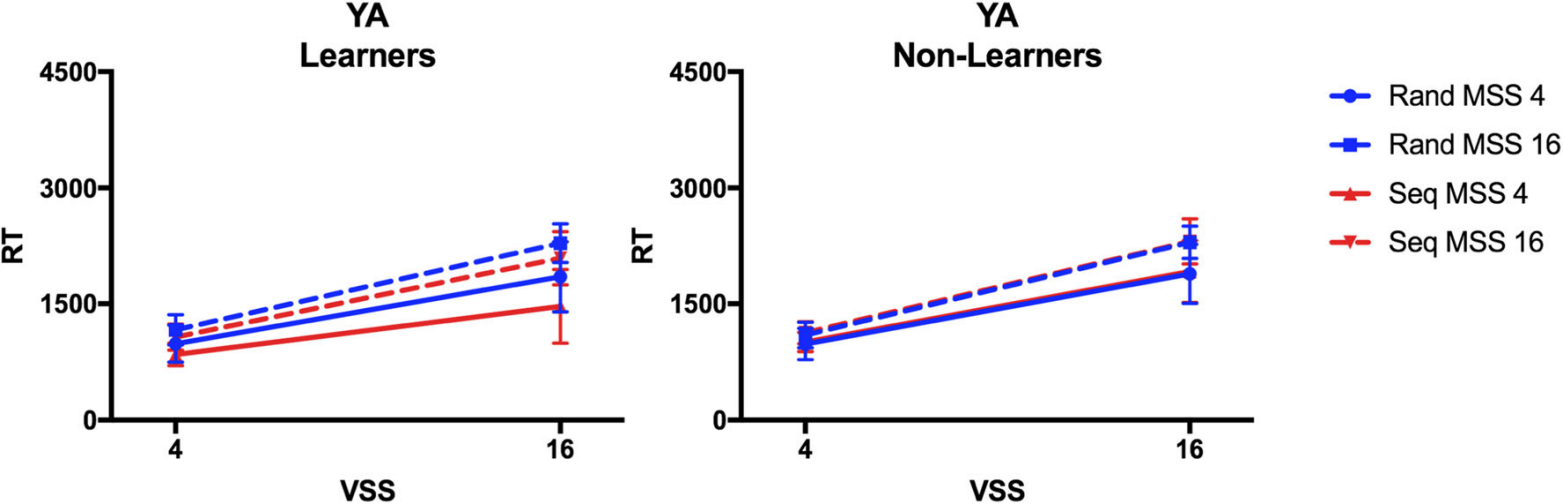
Reaction times (RTs) in Experiment 1 for young learners and non-learners. Mean RTs are plotted for younger adults (YA), split into sub-groups of participants who acquired explicit knowledge about the sequence (Learners) and those who did not (Non-Learners). RTs are plotted as a function of visual set size (VSS) comparing the blocks in which targets appeared in a repeating sequence (Seq) and in which targets appeared randomly (Rand) across trials for smaller and larger memory set sizes (MSS). Error bars indicate standard error of the mean

Together, the results suggest little evidence for sequence-learning effects in hybrid search. In the younger sample only, a small, non-significant RT effect was observable. We followed up on this potential trend by splitting the younger adults into learners and non-learners based on the post-experimental tests of explicit knowledge about the sequence. We defined observers as “Learners” if they reached 75% correct responses on the 2AFC test and/or reported the sequence in the questionnaire. Learners constituted 10 out of 24 younger participants. In the older sample, only one participant scored 75% in the 2AFC test and none reported the sequence in the questionnaire. We compared learning effects between young learners and non-learners in a four-way ANOVA with the factors Learner (learners, non-learners), Visual Set Size (4, 16), Memory Set Size (4, 16), and Target Order (random, sequence). The results are shown in Fig. 4. Besides the significant effects of Visual Set Size, Memory Set Size and the factors’ interaction (all F(1,22)>42.30, all p<.001, all η_p_^2^>.66, BF_10_>86815.31), the ANOVA also revealed a trend-significant effect of Target Order (F(1,22)=4.04, p=.06, η_p_^2^=.16, BF_10_=1.00), and, importantly, a significant interaction of Learner and Target Order (F(1,22)=8.46, p=.008, η_p_^2^=.28, BF_10_=126.66). The learners’ RTs were faster in the sequence relative to random condition (F(1,9)=9.63, p=.01, η_p_^2^=.52, BF_10_=274.33), while non-learners showed no significant sequence effect on RT (F(1,13)=2.19, p=.16, η_p_^2^=.15, BF_01_=3.44). Recall, however, that our calculated power to find this type of interaction was only 0.6 meaning that we should be cautious in interpreting the effect. In addition, the Visual Set Size × Target Order interaction reached significance (F(1,22)=4.74, p=.04, η_p_^2^=.18), though it was not supported by the BF (BF_10_=0.41), reflecting steeper search slopes in the random than in the sequence condition.

Our results suggest that sequence learning can facilitate hybrid search, but only for a subset of observers, who had acquired some explicit knowledge of the repeating target sequence. Thus, there is an important difference between learning mechanisms in our hybrid search task compared to the SRTT and other implicit learning tasks, in which RT benefits also occurred without awareness of the sequential pattern (Dennis et al., 2006; Howard & Howard, 1997; Nissen & Bullemer, 1987). In this hybrid search task, at least, explicit knowledge of the target sequence seems to be necessary for improving performance. For the present task with only 20 repetitions of the target identity, we assume that explicitly represented and retrievable target-order associations have helped the learners to improve their search in the sequence condition. Of course, it is possible that under different task conditions, implicit sequence learning might support hybrid search, but we do not see evidence here (see *General discussion* below).

The ability to acquire explicit knowledge about a target sequence in hybrid search under incidental learning conditions seems to differ between younger individuals and to become even less likely in older age. It remains unclear, however, if older adults could also benefit from the sequence, given explicit knowledge was made available to all observers. We tested this possibility in Experiment 2.

### Experiment 2: Intentional learning

In the second experiment, all observers had explicit knowledge about the sequential target order. Before the experiment, observers were told whether an experimental block would contain a repeating target sequence or whether targets would occur in random order. Previous research on the SRTT showed that explicit sequence knowledge has a positive effect on sequence learning particularly for deterministic sequence structures (Cleeremans & Jimenez, 1998; Frensch & Miner, 1994), as the present one. Assuming that explicit learning underlies sequence effects in hybrid search, we expected that under such intentional learning conditions, the RT benefit in sequence compared to random blocks would now be pronounced across all observers.

**Fig. 5:**
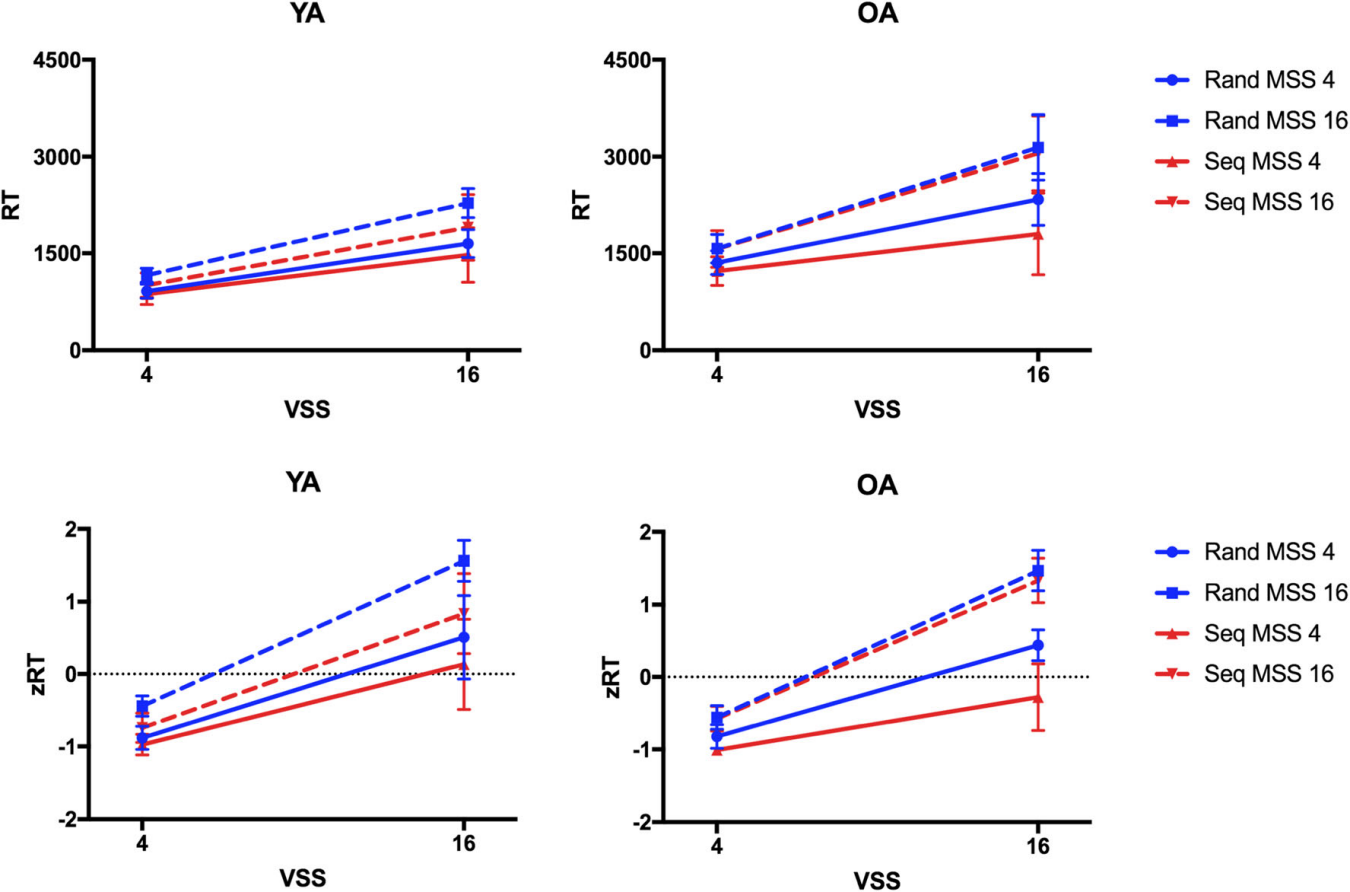
Reaction times (RTs) and z-transformed RT (zRTs) in Experiment 2. Mean RTs and zRTs are plotted for younger adults (YA) and older adults (OA) as a function of visual set size (VSS) comparing the blocks in which targets appeared in a repeating sequence (Seq) and in which targets appeared randomly (Rand) across trials for smaller and larger memory set sizes (MSS). Error bars indicate standard error of the mean

The results, shown in Fig. 5, do indeed indicate a learning effect in Experiment 2, observable in both age groups. As would be expected, the initial four-way ANOVA on RT revealed a main effect of Age (F(1,21)=30.78, p<001, η_p_^2^=.59, BF_10_=983.52) and a main effect of Target Order (F(1,21)=12.60, p=.003, η_p_^2^=.38, BF_10_=348.55). Across all younger and older participants, responses were faster in sequence compared to random blocks. Furthermore, Target Order interacted with Visual Set Size (F(1,21)=8.45, p=009, η_p_^2^>.29, BF_10_=1.40) such that search slopes were shallower in the sequence condition. Target Order did not interact with Memory Set Size (F(1,21)=1.11, p=.30, η_p_^2^=.05, BF_01_=2.60). The three-way interaction between Target Order, Memory Set Size, and Age and the four-way interaction between Target Order, Visual Set Size, Memory Set Size, and Age were significant (both F(1,21)>6.99, p<.02, η_p_^2^>.24). However, Bayes factors were equivocal (BF_10_=2.76 and BF_10_=0.687). Besides, main effects and the interactions of Visual Set Size and Memory Set Size were significant (all F(1,21)>93.49, p<.001, η_p_^2^>.81, BF_10_>88446.51).

The same ANOVA on zRT also showed the significant main effect of Target Order (F(1,21)=13.72, p=.001, η_p_^2^=.40, BF_10_=3.62e+6) and significant interactions between Target Order and Visual Set Size (F(1,21)=8.99, p=.007, η_p_^2^=.30, BF_10_=7.09), between Target Order, Memory Set Size and Age (F(1,21)=12.99, p=.002, η_p_^2^=.38, BF_10_=39.20), and Target Order, Visual Set Size, Memory Set Size, and Age (F(1,21)=6.99, p=.015, η_p_^2^=.25, BF_10_=2.57). The main effect of Age was not significant (F(1,21)=0.001, p=.97, η_p_^2^<.001, BF_01_=4.72). The two-way interactions of Age and Visual Set Size and of Age and Memory Set Size were not significant (both F(1,21)<2.87, p>.10, η_p_^2^<.12, BF_01_>4.01). The three-way interaction between all factors was significant, but not supported by the Bayesian analysis (F(1,21)=7.67, p=.01, η_p_^2^=.26, BF_01_=6.26).

Separate ANOVAs on RT for the two age groups showed a main effect of Target Order (F(1,11)=, p=.04, η_p_^2^=.33, BF_10_=43.94), but no evidence for an interaction between Target Order and Memory Set Size (F(1,11)=2.44, p=.15, η_p_^2^=.18, BF_01_=1.56) in younger adults. This indicates that the learning effect was of similar magnitude for smaller and larger memory sets, though Fig. 5 may suggest that the learning effect was slightly larger in the block with the larger memory set of 16 targets. For older adults, in addition to the main effect of Target Order (F(1,10)=7.63, p=.02, η_p_^2^=.43, BF_10_=16.12), the interaction of Target Order and Memory Set Size was significant (F(1,10)=17.56, p=.002, η_p_^2^=.64, BF_10_=2.51). Older adults showed faster RTs in sequence compared to random blocks only in the block with the smaller memory set (t(10)=4.24, p=.005, *d*=1.33, BF_10_=171.92), but no RT benefit in the block with the larger memory set (t(10)=0.81, p>.99, *d*=0.18, BF_01_=2.92).

**Fig. 6:**
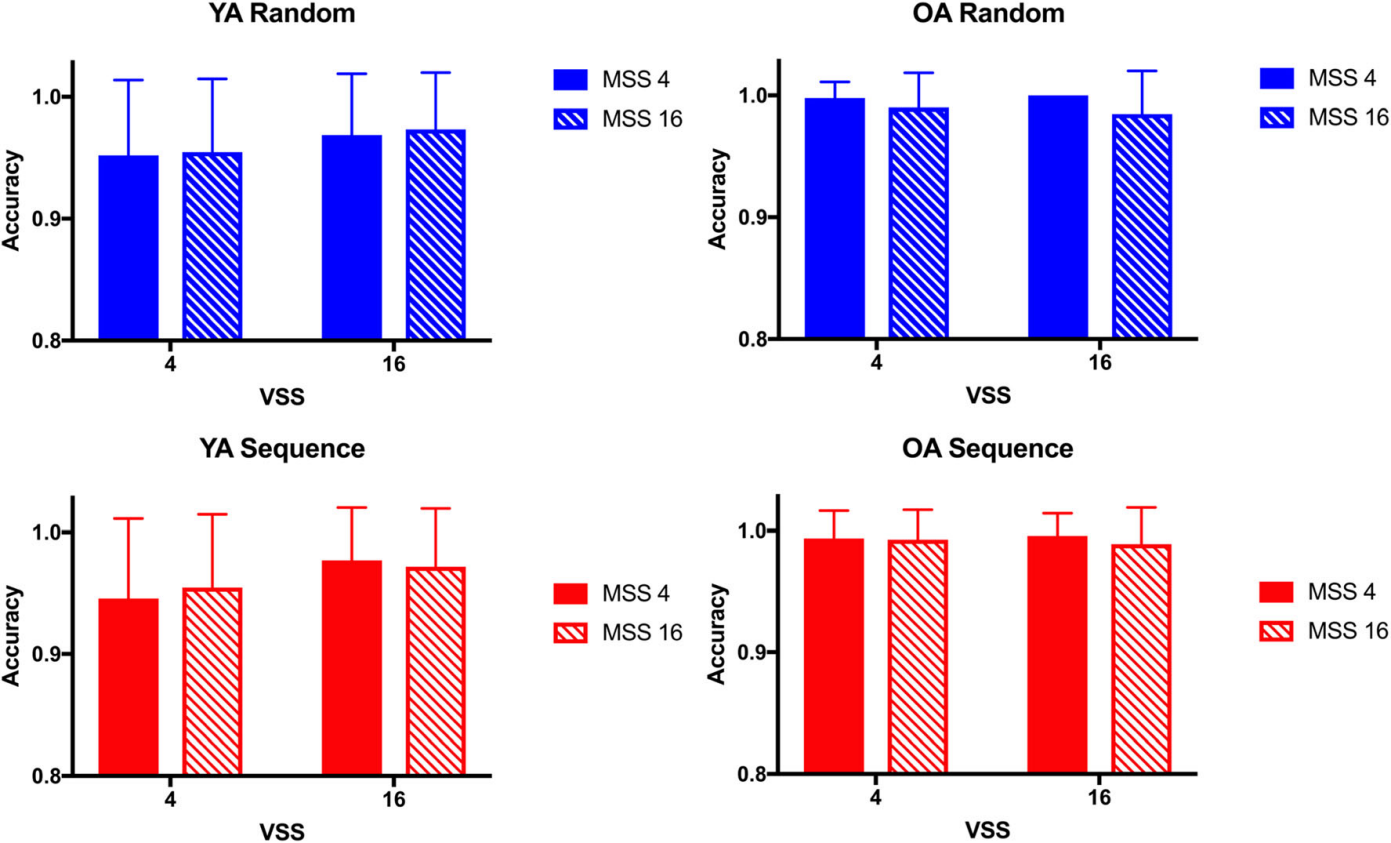
Accuracy (rate of correct clicks) in Experiment 2. Mean accuracy is plotted for younger adults (YA, left) and older adults (OA, right), comparing the blocks in which targets appeared in a repeating sequence (red bars, lower panels) and in which targets appeared randomly (blue bars, upper panels), for smaller and larger visual set sizes (VSS) and memory set sizes (MSS). Error bars indicate standard error of the mean

Figure 6 shows the accuracy data for the different age groups and experimental conditions in Experiment 2. The ANOVA on arcsine-transformed accuracy data revealed a significant main effect of Age (F(1,21)>14.21, p=.001, η_p_^2^>.40, BF_10_=28.78). As in Experiment 1, indicative of a modest speed-accuracy trade-off, younger adults made more errors that older adults (3.7% vs. 1.0%). The main effects of Memory Set Size and of Visual Set Size were significant (both F(1,21)>4.80, p<.05, η_p_^2^>.18, BF_10_>5.19). Across age groups, performance was slightly better in the conditions with larger visual set sizes and smaller memory set sizes. None of the two- and three-way interactions were significant. Importantly, as in Experiment 1, there was no main effect nor interaction involving Target Order (all F(1,21)<1.04, all p>.31, all η_p_^2^<.02, all BF_01_>0.51).

The average performance in the explicit sequence knowledge test was 88% and 86% for younger and older adults, respectively, and, thus, clearly better than chance level (t(22)=8.38, p<.001, *d*=1.75, BF_10_=507002.79). Performance was better for the shorter sequence of four targets (94% and 91% for younger and older adults, respectively) than for the longer sequence of 16 targets (81% and 82% for younger and older adults, respectively). The ANOVA revealed a trend for an effect of Memory Set Size (F(1,21)=3.82, p=.06, η_p_^2^=.15, BF_10_=1.39). There was no effect of Age (F(1,21)=0.02, p=.90, η_p_^2^<.001, BF_01_=2.43) and no interaction of Age and Memory Set Size (F(1,21)=0.10, p=.76, η_p_^2^=.005, BF_01_=4.40). This supports the conclusion that both age groups acquired comparable explicit knowledge about shorter and longer target sequences.

First, the results of Experiment 2 demonstrate that observers acquired and could use the advance knowledge about the presence of a sequence to improve their search performance. In both age groups, we observed explicit learning effects, manifesting as a decrease in RT in the sequence compared to the random condition. This suggests that younger as well as older observers can use a voluntary, top-down mechanism to deploy their knowledge of the sequence during hybrid search. Second, our results revealed an interesting age difference. While younger adults showed a learning effect for both smaller and larger memory set sizes, older adults only benefitted from the sequence if the number of targets to be held in memory was relatively small. Thus, it is possible that a load-dependent limit of explicit sequence-learning effects on hybrid search is sensitive to aging processes.

## General discussion

The present study investigated sequence-learning effects in younger and older adults in a hybrid visual and memory search task with photo-realistic objects. In this task, observers look for one out of multiple target objects among distractor objects. We tested whether hybrid search could be facilitated if targets from the memory set were presented in a repeating sequence over trials.

### Explicit sequence learning facilitates hybrid search

Our results show that sequence learning can improve hybrid search. RTs were faster when targets occurred in a repeating sequential order compared to when targets changed randomly over search trials. Importantly, however, we only found RT benefits when observers knew about the sequence. Under incidental learning conditions in Experiment 1, only about a third of the younger observers showed an RT benefit and those were the observers who had become explicitly aware of the repeating pattern. In Experiment 2, when observers were explicitly told about the presence of a sequential order of targets, a reliable learning effect became manifest across the entire sample. Thus, sequence-learning effects in hybrid search appear to be different from those in SRTTs and other implicit learning tasks, where sequence-based RT benefits are observable independently of the participants’ awareness of the sequential rule (Seger, 1994). Instead, in hybrid search, the sequential order of targets must be learned and represented explicitly in order to show an effect. This process is assumed to draw on limited resources (Frensch & Rünger, 2003) and appears to be affected by normal aging.

What mechanism might underlie the explicit sequence-learning effect in hybrid search? Observers may have formed a mental representation of the sequence structure by learning consecutive target-target associations. After they learned this structure, the current target could serve as a retrieval cue for the next. This predictive cue would allow the observer to pre-activate the expected target’s memory representation as a search template and thus, guide visual attention to the target’s features, making search faster and more efficient. Similarly, endowing observers with target knowledge by pre-cuing its feature or identity has been shown to facilitate visual search in a top-down manner (Anderson et al., 2010; Wolfe, Horowitz, Kenner, Hyle, & Vasan, 2004).

Previous research on hybrid search showed that observers do not easily restrict their memory search in tasks using cues. In studies by Boettcher et al. (2018) and Wiegand and Wolfe (2020), younger and older adults learned explicit target-context associations before a search task. Eight target objects were associated with one context, and eight different target objects with another. Observers recognized the learned associations with high accuracy. However, during search, they did not use the context as a cue to restrict their memory search to the subsets of eight targets associated with this particular context. Instead, RTs indicated that observers searched through the entire target set of 16 objects, at least when context cues switched from trial to trial. Presumably, the flexible retrieval of associations was, though possible, too effortful to be an effective strategy for refining their search. By contrast, the present study shows that explicit sequence knowledge containing target-target associations is more readily applied to ease memory search.

While we found evidence that explicit knowledge was the main driver of our sequence-learning effects on RT in hybrid search, learning effects in the classic SRTT have been typically attributed to implicit processes. In the present experiments, the response is different from the original version of the SRTT. Here the localization of the target on the screen by mouse click, was independent of the regularity in the visual stimulus, i.e., the target identity. Thus, our tasks fall into the category of “pure” perceptual learning tasks (Remillard, 2003). Effects of pure perceptual sequence learning were previously shown to be smaller and less reliable compared to paradigms in which the stimulus and response repetitions are mapped (Abrahamse, et al., 2010; Haider et al., 2013). Consistent with the present finding, some authors have argued that pure perceptual learning relies on explicit awareness and attention (Hikosaka et al., 1999; Willingham, 1999). However, others have reported perceptual sequence learning in the absence of explicit knowledge about the sequential structure (Mayr, 1996) and have argued that similar mechanisms underlie the implicit learning of perceptual and response regularities (Remillard, 2011). One critical determinant of whether or not implicit learning occurs in a perceptual sequence learning might be the feature that carries the regularity information. In the majority of studies that reported perceptual learning effects, this was the target location (Deroost & Coomans, 2018; Deroost & Soetens, 2006a, 2006b; Mayr, 1996; Marcus, Karatekin, & Markiewicz, 2006; Lum, 2020; Remillard, 2003, 2011). It has been suggested that spatial structures are learned more effectively than non-spatial regularities (Koch & Hoffmann, 2000). Moreover, it has been argued that the sequential order of oculo-motor movements may support implicit learning in SRTTs with repeating target locations (Deroost & Coomans, 2018).

In the present hybrid search task, we tested whether the sequence of the target identities would be learned, independently of both the spatial location of the target and the response. It is entirely possible that the sequence might have been learned more easily and implicitly, if the targets would have appeared at repeating instead of random positions. However, our focus was to investigate sequence-learning effects on memory retrieval in visual search, independent of the target location in space. Our results suggest that this learning effect was supported by explicit representations of the sequence of target identities.

We performed additional analyses on repetition effects to investigate the time course of learning (see Online Supplementary Material II). Those demonstrated the expected practice effects on RTs but also showed that learning effects barely increased over time. Thus, the proposed associative target structure in the hybrid search task must have been established quickly within one or very few exposures. This supports our assumption that the sequence-learning effects we observed in this hybrid search task rely on an explicitly-accessed associative episodic memory trace, which can be rapidly established within a single learning episode (Gallo & Wheeler, 2013). By contrast, implicit learning effects have been found to increase gradually with the number of repetitions (Mayr, 1996; Nissen & Bullemer, 1987; Reber, 1989). It is possible that we did not observe incidental learning effects without sequence awareness in Experiment 1 because 20 repetitions were simply not enough to implicitly learn the sequence. In the original SRTT by Nissen and Bullemer (1987), learning effects of ten-element sequences were observable after only a few repetitions and increased further over ten blocks of ten repetitions each. However, this relatively quick acquisition of implicit sequence was found in studies with mapped stimulus-response regularities and more repetitions might be required in perceptual learning tasks without spatial regularities like ours (Koch & Hoffmann, 2000). Thus, although the analyses of repetition effects did not even show a trend towards increasing learning effects over repetitions, we would not exclude that implicit learning may have occurred after many more exposures. At the very least, however, we can say that implicit learning in hybrid search does not happen quickly and explicit learning effects drive the RT benefit we observed here.

### Age-related decline in explicit sequence learning in hybrid search

With regard to adult age differences, the first important finding from Experiment 1 was that older adults are (even) less likely than younger adults to acquire explicit knowledge about a target sequence in hybrid search. We propose that the young learners in Experiment 1 may have built up a global episodic task representation that enabled them to notice the pattern. The transition from incidental learning to explicit awareness involves forming an integrated memory representation of attended task aspects and is governed by control functions (Cleeremans & Jimenez, 2002; Niv, 2019). Additionally, detection of the hidden rule under incidental learning conditions further requires a form of metacognitive control, which is highly variable among individuals and decreases with older age (Hertzog, 2016; Rose et al., 2010; Souchay & Isingrini, 2004). The contribution of executive control functions to individual and age differences in explicit learning is supported by previous findings from the SRTT, where older adults were less likely than younger adults to acquire explicit sequence knowledge incidentally (Howard & Howard, 1997).

Experiment 2, however, demonstrates that explicit learning of a target sequence under intentional learning instruction is preserved in older age. When told about the sequence, also older adults were able to use this knowledge to improve search. Previous research has shown that intentional instructions can affect implicit learning negatively, particularly in older adults (Howard & Howard, 2001). However, if the learning effect on RT is driven by explicit sequence knowledge, as in the present search task, younger and older adults both show learning benefits. These results are therefore consistent with the finding that conscious expectation of target-relevant features effectively facilitates visual search in older adults (Madden et al., 2007). However, we also found an interaction between learning, memory load and age. While younger adults showed a sequence-learning effect in blocks with both four and 16 to-be-memorized targets, older adults only benefitted from the sequential target order when the memory load was lower. Although we are careful in drawing strong conclusions from this effect given the relatively small sample size, our data suggest that cognitive and neural resource limitations in older age (Park & Reuter-Lorenz, 2009) may affect the acquisition and usage of sequence knowledge in complex cognitive tasks. In accordance with this interpretation, age differences in sequence learning under explicit and intentional learning conditions have been previously shown (Frensch & Miner, 1994; Howard & Howard, 2001) and were attributed to age-related decline in central executive functions (Salthouse et al., 2003; Unsworth & Engle, 2005).

Age differences were also found in more difficult SRTTs, such as learning higher-order sequences (Curran, 1997), or when learning was embedded in a dual-task (Vandenbossche et al., 2014). In load manipulation, used in the present studies, we did not change the simple first-order deterministic sequence structure. Studies demonstrated that older adults show impairments in associative memory for temporal order (Cerella et al., 2006; Czernochowski et al., 2008; Naveh-Benjamin, 2000) and executive attentional control needed for organizing long-term memory contents (Moscovitch, 1992; Posner & Snyder, 1975; Rosen et al., 2016; Schneider & Shiffrin, 1977). We presume that the task-load dependent age differences in sequence-learning effects in hybrid search could also be attributed to this general age-related decline in attention and memory functions due to predominant changes in the prefrontal and medial temporal lobes (Grady, 2008; Wang & Cabeza, 2017; Zanto & Gazzaley, 2017), which impacts learning under cognitively challenging conditions (Schendan et al., 2003). More specifically, in the easier blocks with only four target objects, older adults may have been able to compensate for the deficit in building up persistent long-term memory temporal-order associations between target objects by instead actively holding the targets in working memory during the search task (Schneider-Garces et al., 2010). Obviously, however, this strategy is impractical when the load exceeds the limits of working memory as in the difficult block with 16 targets, which was reflected in their RTs. Interestingly, we did not find any age differences in explicit knowledge about the target sequence in the AFC test following the experimental task, neither for the block with smaller nor larger memory set sizes. Thus, the age deficit appears not to affect the retrieval of targets per se, but to occur specifically when the sequence knowledge is applied in the search context.

### Limitations and future directions

Though the study was adequately powered for the main questions of interest, the sample sizes in the present experiments were rather small. In order to substantiate our conclusions, a replication and extension of the results in future studies with larger samples is therefore desirable. Specifically, given that explicit learning appears to be key to benefiting from a sequential target structure in the present task version, the individual and age differences in extracting the structure incidentally are compelling and worth further exploration. In our sample group of Experiment 1 (incidental learning), the young learners scored higher than the non-learners in the DSST, a neuropsychological test of visuo-motor speed. In future studies with larger samples, it would be interesting to examine the relationship between explicit sequence learning in hybrid search and executive functions or general fluid intelligence systematically (Unsworth & Engle, 2005). Furthermore, it would be interesting to test whether the skill is transferable to other explicit learning tasks with ecologically relevant sequences, such as remembering a route for navigation or second language acquisition.

Second, as noted above, it is possible that implicit learning of a target sequence could facilitate hybrid search under different task settings than those in the present study. In future studies, to promote implicit learning, one could use probabilistic rather than deterministic sequences (Cleeremans & Jimenez, 1998; Jiménez, Vaquero, & Lupiánez, 2006) and a larger number of repetitions over multiple learning blocks. In addition, both implicit and explicit sequence knowledge should be assessed using a process dissociation procedure (Jacoby, 1991; Destrebecqz & Cleeremans, 2001).

Finally, here, we would only be speculating on the neuro-cognitive mechanisms underlying the age-related changes in learning we observed behaviorally. Combining the hybrid search task with neuroimaging or electrophysiology (Drew et al., 2019; Williams et al., 2020) would be important for investigating these underlying cognitive, strategic and neural changes further and integrate our results into neurocognitive theories of aging (Dennis & Cabeza, 2011).

## Supplementary Information

ESM 1 (DOCX 29849 kb)

ESM 2 (DOCX 21 kb)

## Footnotes

1 Cognitive reserve refers to an individual’s resilience to brain damage (Stern, 2002). It describes the phenomenon where older adults with more cognitively stimulating environments (e.g., longer education, challenging occupation, leisure and social activities) show less age-related cognitive decline

## References

[CR1] Abrahamse EL, Jiménez L, Verwey WB, Clegg BA (2010). Representing serial action and perception. Psychonomic Bulletin & Review.

[CR2] Allen TA, Morris AM, Stark SM, Fortin NJ, Stark CE (2015). Memory for sequences of events impaired in typical aging. Learning & Memory.

[CR3] Anderson GM, Heinke D, Humphreys GW (2010). Featural guidance in conjunction search: The contrast between orientation and color. Journal of Experimental Psychology: Human Perception and Performance.

[CR4] Ashe J, Lungu OV, Basford AT, Lu X (2006). Cortical control of motor sequences. Current Opinion in Neurobiology.

[CR5] Bailey IL, Lovie JE (1976). New design principles for visual acuity letter charts. American Journal of Optometry and Physiological Optics.

[CR6] Blair JR, Spreen O (1989). Predicting premorbid IQ: a revision of the National Adult Reading Test. The Clinical Neuropsychologist.

[CR7] Boettcher SE, Drew T, Wolfe JM (2018). Lost in the supermarket: Quantifying the cost of partitioning memory sets in hybrid search. Memory & Cognition.

[CR8] Brady TF, Konkle T, Alvarez GA, Oliva A (2008). Visual long-term memory has a massive storage capacity for object details. Proceedings of the National Academy of Sciences.

[CR9] Brainard DH (1997). The psychophysics toolbox. Spatial Vision.

[CR10] Broadbent DE, Cooper PF, FitzGerald P, Parkes KR (1982). The cognitive failures questionnaire (CFQ) and its correlates. British Journal of Clinical Psychology.

[CR11] Cabeza R, Anderson ND, Houle S, Mangels JA, Nyberg L (2000). Age-related differences in neural activity during item and temporal-order memory retrieval: a positron emission tomography study. Journal of Cognitive Neuroscience.

[CR12] Cerella J, Onyper SV, Hoyer WJ (2006). The associative-memory basis of cognitive skill learning: Adult age differences. Psychology and Aging.

[CR13] Cherry KE, Stadler ME (1995). Implicit learning of a nonverbal sequence in younger and older adults. Psychology and Aging.

[CR14] Chun MM, Jiang Y (1998). Contextual cueing: Implicit learning and memory of visual context guides spatial attention. Cognitive Psychology.

[CR15] Cleeremans, A., & Jiménez, L. (1998). Implicit sequence learning: The truth is in the details. *Handbook of implicit learning* (pp. 323–364).

[CR16] Cleeremans A, Jiménez L (2002). Implicit learning and consciousness: A graded, dynamic perspective. Implicit learning and consciousness.

[CR17] Craik FIM, Jennings JM, Craik FIM, Salthouse TA (1992). Human memory. The handbook of aging and cognition.

[CR18] Curran T (1997). Effects of aging on implicit sequence learning: Accounting for sequence structure and explicit knowledge. Psychological Research.

[CR19] Czernochowski D, Fabiani M, Friedman D (2008). Use it or lose it? SES mitigates age-related decline in a recency/recognition task. Neurobiology of Aging.

[CR20] Dennis NA, Cabeza R (2011). Age-related dedifferentiation of learning systems: an fMRI study of implicit and explicit learning. Neurobiology of Aging.

[CR21] Dennis NA, Howard JH, Howard DV (2006). Implicit sequence learning without motor sequencing in young and old adults. Experimental Brain Research.

[CR22] Deroost N, Soetens E (2006). Perceptual or motor learning in SRT tasks with complex sequence structures. Psychological Research.

[CR23] Deroost N, Soetens E (2006). Spatial processing and perceptual sequence learning in SRT tasks. Experimental Psychology.

[CR24] Deroost N, Coomans D (2018). Is sequence awareness mandatory for perceptual sequence learning: An assessment using a pure perceptual sequence learning design. Acta Psychologica.

[CR25] Destrebecqz A, Cleeremans A (2001). Can sequence learning be implicit? New evidence with the process dissociation procedure. Psychonomic Bulletin & Review.

[CR26] Drew T, Williams LH, Wolfe JM, Wiegand I (2019). How do you know if you saw that? Electrophysiological correlates of searching through memory. Journal of Vision.

[CR27] Faul F, Erdfelder E, Lang AG, Buchner A (2007). G* Power 3: A flexible statistical power analysis program for the social, behavioral, and biomedical sciences. Behavior Research Methods.

[CR28] Faust ME, Balota DA, Spieler DH, Ferraro FR (1999). Individual differences in information-processing rate and amount: implications for group differences in response latency. Psychological Bulletin.

[CR29] Folstein MF, Folstein SE, McHugh PR (1975). ‘Mini Mental State’. A practical method for grading the cognitive state of patients for the clinician. Journal of Psychiatric Research.

[CR30] Frensch PA, Miner CS (1994). Effects of presentation rate and individual differences in short-term memory capacity on an indirect measure of serial learning. Memory & Cognition.

[CR31] Frensch PA, Rünger D (2003). Implicit learning. Current Directions in Psychological Science.

[CR32] Gallo DA, Wheeler ME, Reisberg D (2013). Episodic memory. Oxford library of psychology. The Oxford handbook of cognitive psychology.

[CR33] Grady CL (2008). Cognitive neuroscience of aging. Annals of the New York Academy of Sciences.

[CR34] Goschke T, Stadler MA, Frensch PA (1998). Implicit learning of perceptual and motor sequences: Evidence for independent learning systems. Handbook of implicit learning.

[CR35] Goschke T, Bolte A (2012). On the modularity of implicit sequence learning: Independent acquisition of spatial, symbolic, and manual sequences. Cognitive Psychology.

[CR36] Haider H, Eberhardt K, Kunde A, Rose M (2013). Implicit visual learning and the expression of learning. Consciousness and Cognition.

[CR37] Hertzog C, Dunlosky J, Tauber SK (2016). Aging and metacognitive control. Oxford library of psychology. The Oxford handbook of metamemory.

[CR38] Hikosaka O, Nakahara H, Rand MK, Sakai K, Lu X, Nakamura K, Doya K (1999). Parallel neural networks for learning sequential procedures. Trends in Neurosciences.

[CR39] Hogg RV, Craig AT (1995). Introduction to mathematical statistics.

[CR40] Howard DV, Howard JH (1989). Age differences in learning serial patterns: direct versus indirect measures. Psychology and Aging.

[CR41] Howard JHJ, Howard DV (1997). Age differences in implicit learning of higher order dependencies in serial patterns. Psychology and Aging.

[CR42] Howard DV, Howard JH (2001). When it does hurt to try: Adult age differences in the effects of instructions on implicit pattern learning. Psychonomic Bulletin & Review.

[CR43] Howard JH, Howard DV, Dennis NA, Yankovich H, Vaidya CJ (2004). Implicit spatial contextual learning in healthy aging. Neuropsychology.

[CR44] Howard DV, Howard JH, Japikse K, DiYanni C, Thompson A, Somberg R (2004). Implicit sequence learning: effects of level of structure, adult age, and extended practice. Psychology and Aging.

[CR45] Ishihara, I. (1980). *Ishihara's tests for color-blindness* (Concise ed.). Tokyo: Kanehara & Co., LTD.

[CR46] Jacoby LL (1991). A process dissociation framework: Separating automatic from intentional uses of memory. Journal of Memory and Language.

[CR47] Jiménez L, Vaquero JM, Lupiánez J (2006). Qualitative differences between implicit and explicit sequence learning. Journal of experimental psychology: Learning, Memory, and Cognition.

[CR48] Kahana MJ (1996). Associative retrieval processes in free recall. Memory & Cognition.

[CR49] Kass RE, Raftery AE (1995). Bayes factors. Journal of the American Statistical Association.

[CR50] Keele S, Ivry RB, Mayr U, Hazeltine E, Heuer H (2003). The cognitive and neural architecture of sequence representation. Psychological Review.

[CR51] Koch I, Hoffmann J (2000). The role of stimulus-based and response-based spatial information in sequence learning. Journal of Experimental Psychology: Learning, Memory, & Cognition.

[CR52] Koch I, Blotenberg I, Fedosejew V, Stephan DN (2020). Implicit perceptual learning of visual-auditory modality sequences. Acta Psychologica.

[CR53] Konkle T, Brady TF, Alvarez GA, Oliva A (2010). Conceptual distinctiveness supports detailed visual long-term memory for real-world objects. Journal of Experimental Psychology: General.

[CR54] Lashley KS, Jeffress LA (1951). The problem of serial order in behavior. Cerebral mechanisms in behavior: The Hixon symposium.

[CR55] Lum JAG (2020). Incidental learning of a visuo-motor sequence modulates saccadic amplitude: Evidence from the serial reaction time task. Journal of Experimental Psychology: Learning, Memory, and Cognition.

[CR56] Madden DJ, Whiting WL, Spaniol J, Bucur B (2005). Adult age differences in the implicit and explicit components of top-down attentional guidance during visual search. Psychology and Aging.

[CR57] Madden DJ, Spaniol J, Bucur B, Whiting WL (2007). Age-related increase in top-down activation of visual features. Quarterly Journal of Experimental Psychology.

[CR58] Marcus DJ, Karatekin C, Markiewicz S (2006). Oculomotor evidence of sequence learning on the serial reaction time task. Memory & Cognition.

[CR59] Mayr U (1996). Spatial attention and implicit sequence learning: Evidence for independent learning of spatial and nonspatial sequences. Journal of Experimental Psychology: Learning, Memory, and Cognition.

[CR60] McCarley JS, Kramer AF, Colcombe AM, Scialfa CT (2004). Priming of pop-out in visual search: A comparison of young and old adults. Aging, Neuropsychology, and Cognition.

[CR61] Meier B, Cock J, Seel NM (2012). Implicit Sequence Learning. Encyclopedia of the Sciences of Learning.

[CR62] Mitchell DB (1989). How many memory systems? Evidence from aging. Journal of Experimental Psychology: Learning, Memory, and Cognition.

[CR63] Moscovitch M (1992). Memory and working-with-memory: A component process model based on modules and central systems. Journal of Cognitive Neuroscience.

[CR64] Nattkemper D, Prinz W (1997). Stimulus and response anticipation in a serial reaction task. Psychological Research.

[CR65] Naveh-Benjamin M (2000). Adult age differences in memory performance: tests of an associative deficit hypothesis. Journal of Experimental Psychology: Learning, Memory, and Cognition.

[CR66] Negash S, Howard DV, Japikse KC, Howard JH (2003). Age-related differences in implicit learning of non-spatial sequential patterns. Aging, Neuropsychology, and Cognition.

[CR67] Nelson, H. E. (1982). *National Adult Reading Test (NART): For the assessment of premorbid intelligence in patients with dementia: Test manual*. NFER-Nelson.

[CR68] Nilsson LG (2003). Memory function in normal aging. Acta Neurologica Scandinavica.

[CR69] Nissen MJ, Bullemer P (1987). Attentional requirements of learning: Evidence from performance measures. Cognitive Psychology.

[CR70] Niv Y (2019). Learning task-state representations. Nature Neuroscience.

[CR71] Nucci M, Mapelli D, Mondini S (2012). Cognitive Reserve Index questionnaire (CRIq): a new instrument for measuring cognitive reserve. Aging Clinical and Experimental Research.

[CR72] Park DC, Reuter-Lorenz P (2009). The adaptive brain: aging and neurocognitive scaffolding. Annual Review of Psychology.

[CR73] Posner MI, Snyder CRR, RL RLS (1975). Attention and cognitive control. Information processing and cognition: The Loyola symposium.

[CR74] Prull MW, Gabrieli JDE, Bunge SA, Craik FIM, Salthouse TA (2000). Age-related changes in memory: A cognitive neuroscience perspective. The handbook of aging and cognition.

[CR75] Radloff LS (1977). The CES-D scale: A self-report depression scale for research in the general population. Applied Psychological Measurement.

[CR76] Reber AS (1989). Implicit learning and tacit knowledge. Journal of Experimental Psychology: General.

[CR77] Remillard G (2003). Pure perceptual-based sequence learning. Journal of Experimental Psychology: Learning, Memory, and Cognition.

[CR78] Remillard G (2011). Pure perceptual-based learning of second-, third-, and fourth-order sequential probabilities. Psychological Research.

[CR79] Robertson EM, Pascual-Leone A (2001). Aspects of sensory guidance in sequence learning. Experimental Brain Research.

[CR80] Rose M, Haider H, Büchel C (2010). The emergence of explicit memory during learning. Cerebral Cortex.

[CR81] Rosen ML, Stern CE, Michalka SW, Devaney KJ, Somers DC (2016). Cognitive control network contributions to memory-guided visual attention. Cerebral Cortex.

[CR82] Rouder JN, Morey RD, Speckman PL, Province JM (2012). Default Bayes factors for ANOVA designs. Journal of Mathematical Psychology.

[CR83] Rouder JN, Morey RD, Verhagen J, Swagman AR, Wagenmakers EJ (2017). Bayesian analysis of factorial designs. Psychological Methods.

[CR84] Salthouse TA, Atkinson TM, Berish DE (2003). Executive functioning as a potential mediator of age-related cognitive decline in normal adults. Journal of Experimental Psychology: General.

[CR85] Schacter DL (1987). Implicit memory: History and current status. Journal of Experimental Psychology: Learning, Memory, and Cognition.

[CR86] Schendan HE, Searl MM, Melrose RJ, Stern CE (2003). An FMRI study of the role of the medial temporal lobe in implicit and explicit sequence learning. Neuron.

[CR87] Schneider W, Shiffrin RM (1977). Controlled and automatic human information processing: I. Detection, search, and attention. Psychological Review.

[CR88] Schneider-Garces NJ, Gordon BA, Brumback-Peltz CR, Shin E, Lee Y, Sutton BP (2010). Span, CRUNCH, and beyond: working memory capacity and the aging brain. Journal of Cognitive Neuroscience.

[CR89] Schugens MM, Daum I, Spindler M, Birbaumer N (1997). Differential effects of aging on explicit and implicit memory. Aging, Neuropsychology, and Cognition.

[CR90] Schwarb H, Schumacher EH (2012). Generalized lessons about sequence learning from the study of the serial reaction time task. Advances in Cognitive Psychology.

[CR91] Seger CA (1994). Implicit learning. Psychological Bulletin.

[CR92] Shimamura AP (1995). Memory and the Prefrontal Cortex. Annals of the New York Academy of Sciences.

[CR93] Souchay C, Isingrini M (2004). Age related differences in metacognitive control: Role of executive functioning. Brain and cognition.

[CR94] Standing L, Conezio J, Haber RN (1970). Perception and memory for pictures: Single-trial learning of 2500 visual stimuli. Psychonomic Science.

[CR95] Stern Y (2002). What is cognitive reserve? Theory and research application of the reserve concept. Journal of the International Neuropsychological Society.

[CR96] Unsworth N, Engle RW (2005). Individual differences in working memory capacity and learning: Evidence from the serial reaction time task. Memory & Cognition.

[CR97] Vandenbossche J, Coomans D, Homblé K, Deroost N (2014). The effect of cognitive aging on implicit sequence learning and dual tasking. Frontiers in psychology.

[CR98] Wagenmakers EJ, Love J, Marsman M, Jamil T, Ly A, Verhagen J (2018). Bayesian inference for psychology, part II: Example applications with JASP. Psychonomic Bulletin and Review.

[CR99] Wang WC, Cabeza R (2017). Episodic memory encoding and retrieval in the aging brain. In: Cognitive neuroscience of aging: linking cognitive and cerebral aging, 2.

[CR100] Wechsler D (1958). The measurement and appraisal of adult intelligence.

[CR101] Wolfe JM (2012). Saved by a log: How do humans perform hybrid visual and memory search?. Psychological Science.

[CR102] Wolfe JM, Evans KK, Drew T, Aizenman A, Josephs E (2016). How do radiologists use the human search engine?. Radiation Protection Dosimetry.

[CR103] Wolfe J, Horowitz T, Kenner NM, Hyle M, Vasan N (2004). How fast can you change your mind? The speed of top-down guidance in visual search. Vision Research.

[CR104] Wiegand I, Finke K, Müller HJ, Töllner T (2013). Event-related potentials dissociate perceptual from response-related age effects in visual search. Neurobiology of Aging.

[CR105] Wiegand I, Wolfe (2020). Age doesn’t matter much: Hybrid visual and memory search is preserved in older age. Aging, Neuropsychology, & Cognition.

[CR106] Wiegand I, Seidel C, Wolfe J (2019). Hybrid foraging search in younger and older age. Psychology and Aging.

[CR107] Willingham DB, Nissen MJ, Bullemer P (1989). On the development of procedural knowledge. Journal of Experimental Psychology: Learning, Memory, and Cognition.

[CR108] Williams L, Wiegand I, Lavelle M, Wolfe J, Fukuda K, Drew T (2020). What is the role of working memory in hybrid search?: Evidence from the contralateral delay activity. Journal of Vision.

[CR109] Willingham DB (1999). Implicit motor sequence learning is not purely perceptual. Memory & Cognition.

[CR110] Zacks J, Tversky B (2001). Event structure in perception and conception. Psychological Bulletin.

[CR111] Zanto TP, Gazzaley A (2017). Selective attention and inhibitory control in the aging brain. Cognitive neuroscience of aging: Linking Cognitive and Cerebral Aging.

