# Supplementary material for: Order, please! Explicit sequence learning in hybrid search in younger and older age
Source: Mem Cognit. 2021 Apr 19;49(6):1220–35. doi: 10.3758/s13421-021-01157-2 (PMC8313466; doi:10.3758/s13421-021-01157-2)
Supplement: Supplementary file 1 — (DOCX 29849 kb) [file 13421_2021_1157_MOESM1_ESM.docx]

**Supplementary Material II**

**Repetition effects**

As sequence learning effects may evolve over time, we included Repetition as an additional factor in the ANOVA reported in the main paper. We calculated the average RT in four bins according to the number of repetitions of the sequence in the experiment (1-5, 6-10, 11-15, 16-20). In the random condition, the respective trials in which the objects appeared in random order were averaged. This resulted in a 5-way ANOVA with the factors Repetition, Target Order, Visual Set Size, Memory Set Size, and Age. We here report only the main effects and interactions involving the factor Repetition, as all other relevant effects were described in the main text.

**Experiment 1: Incidental Learning**

The ANOVA revealed a main effect of Repetition [F(3,102)=4.372, p=.006, η_p_^2^=.112] and significant 2- and 3-way interactions between Repetition, Visual Set Size, and Memory Set Size [all F(3,102)>3.71, all p<.015]. As visible in Figure S1, over trials in a block, observers became faster, and the set size effects became smaller. This suggests that practice improved hybrid search performance, and practice effects were larger when visual and memory load was high. The Repetition and set size effects did not significantly interact with Age [all F(3,102)<2.55, p>.06, η^2^=.07], suggesting similar practice effects across age groups in Experiment 1. Importantly, interactions involving Repetition and Target Order were non-significant [all F(3,102)<1.88, all p>.13, all η_p_^2^<.06], except one significant interaction between Target Order, Repetition, Memory Set Size and Age [F(3,102)=2.91, p=.04, η_p_^2^=.08]. We followed-up on this interaction with separate ANOVAs for younger and older adults, which revealed for both age groups, main effects of Repetition (YA: F(3,69)=8.277, p<.001, η^2^=.27, OA: F(3,33)=4.728, p=.007, η_p_^2^=.30) and interactions of Repetition and Memory Set Size (YA: F(3,69) =4.761, p=.004, η_p_^2^=.17, OA: F(3,33)=4.00, p=.016, η_p_^2^=.27). RT decreased with repetitions, especially in the high memory load condition. Interactions involving Repetition and Target Order, however, were not significant in either age group (all F <3.5, all p>.07, all η_p_^2^<.14). Notably, also when we included the factor Learner into the ANOVA on YA, we found no significant interactions including Learner, Target Order, and Repetition (all F(3,66)<2.17, p>.10, η_p_^2^<.09)


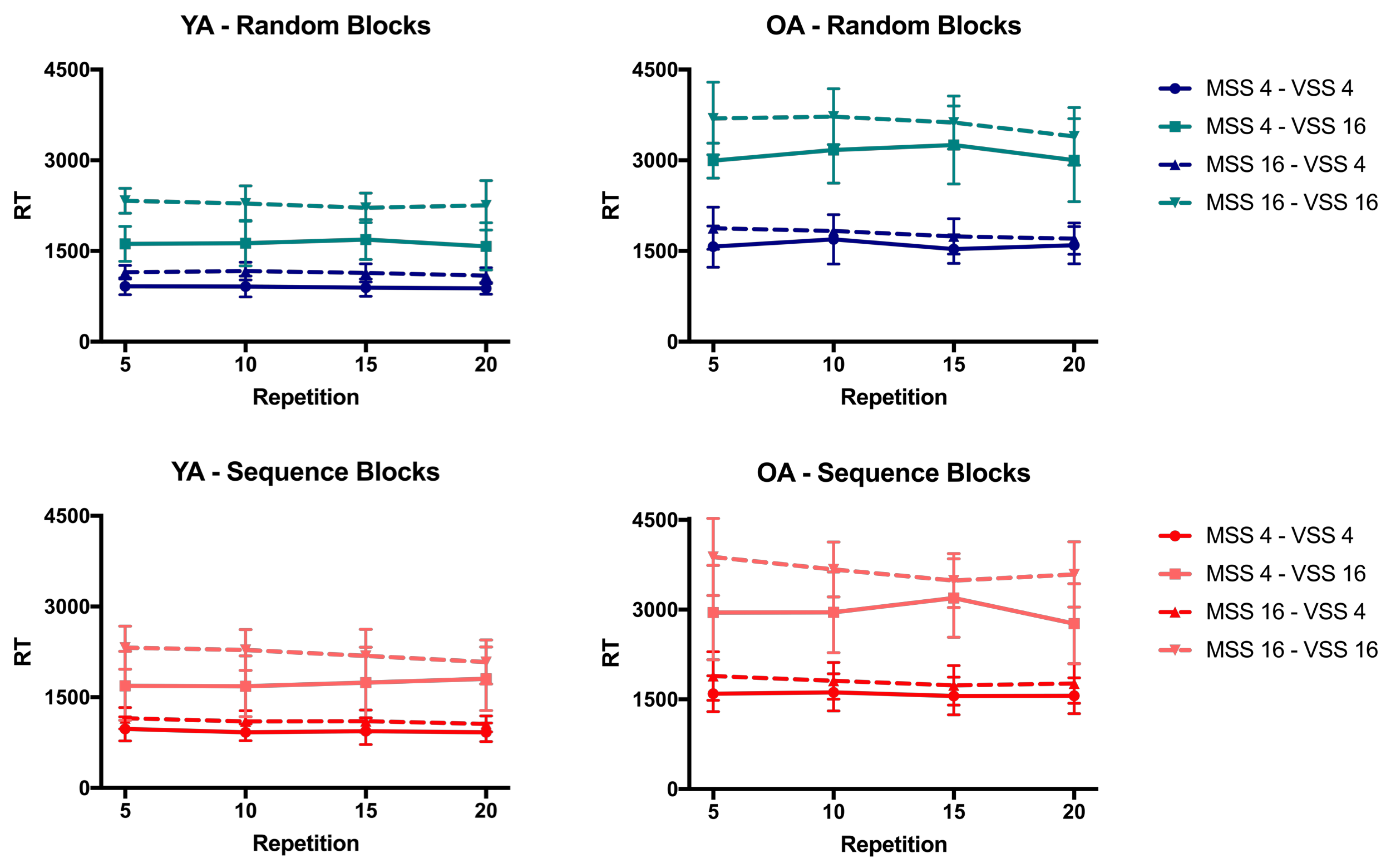


Figure S1: Reaction times (RT) for younger adults (YA) and older adults (OA) are plotted as a function of repetitions, comparing conditions with smaller (4) and larger (16) visual set sizes (VSS) and memory set sizes (MSS) in blocks with random (left) and repeating (right) target sequences.

Our results demonstrate an overall decrease of RT over trials, likely reflecting practice effects. However, there was no repetition effect specific to the sequence learning effect, i.e. the relative RT benefit in sequence versus random blocks did not increase over repetitions. We also did not observe a repetition effect on learning in the subgroup of younger adult learners. As only a few subject showed a sequence learning effect Experiment 1, however, we may have been underpowered to detect a repetition effect in the learning effect on RT

**Experiment 2: Intentional Learning**

The ANOVA revealed a significant main effect of Repetition (F(3,63)=20.30, p<.001, η_p_^2^=.49) and 2-way interactions between Repetition and Visual Set Size, and Repetition and Memory Set Size (both F(3,63)>6.98, p<.001, η_p_^2^>.24). There were further interactions between Repetition and Age (F(3,63)=5.912, p=.001, η_p_^2^=.22) and between Repetition, Memory Set Size and Age (F(3,63)=5.68, p=.002, η_p_^2^=.21).

In younger adults, neither the main effect nor any interactions involving Repetition were significant (all F(3,33)<2.50, p>.07, η_p_^2^<.19). Older adults, by contrast, showed a significant main effect of repetition (F(3,30)=28.84, p<.001, η_p_^2^=.74) as well as significant 2- and 3-way interactions between Repetition, Visual Set Size, and Memory Set Size (all F(3,30)>4.28, p<.02, η_p_^2^>.30). The decrease in RT over time was pronounced at higher visual and memory set sizes. Furthermore, there was a significant interaction between Target Order, Repetition, and Memory Set Size in older adults [F(3,30)=4.88, p=.007, η_p_^2^=.33]. Follow-up ANOVAs on older adults’ RT showed that Repetition interacted significantly with Target Order in the high memory set size condition [F(3,30)=3.66, p=.02, η_p_^2^=.22], but not in the small memory set size condition [F(3,30)=0.87, p=.47, η_p_^2^=.003].


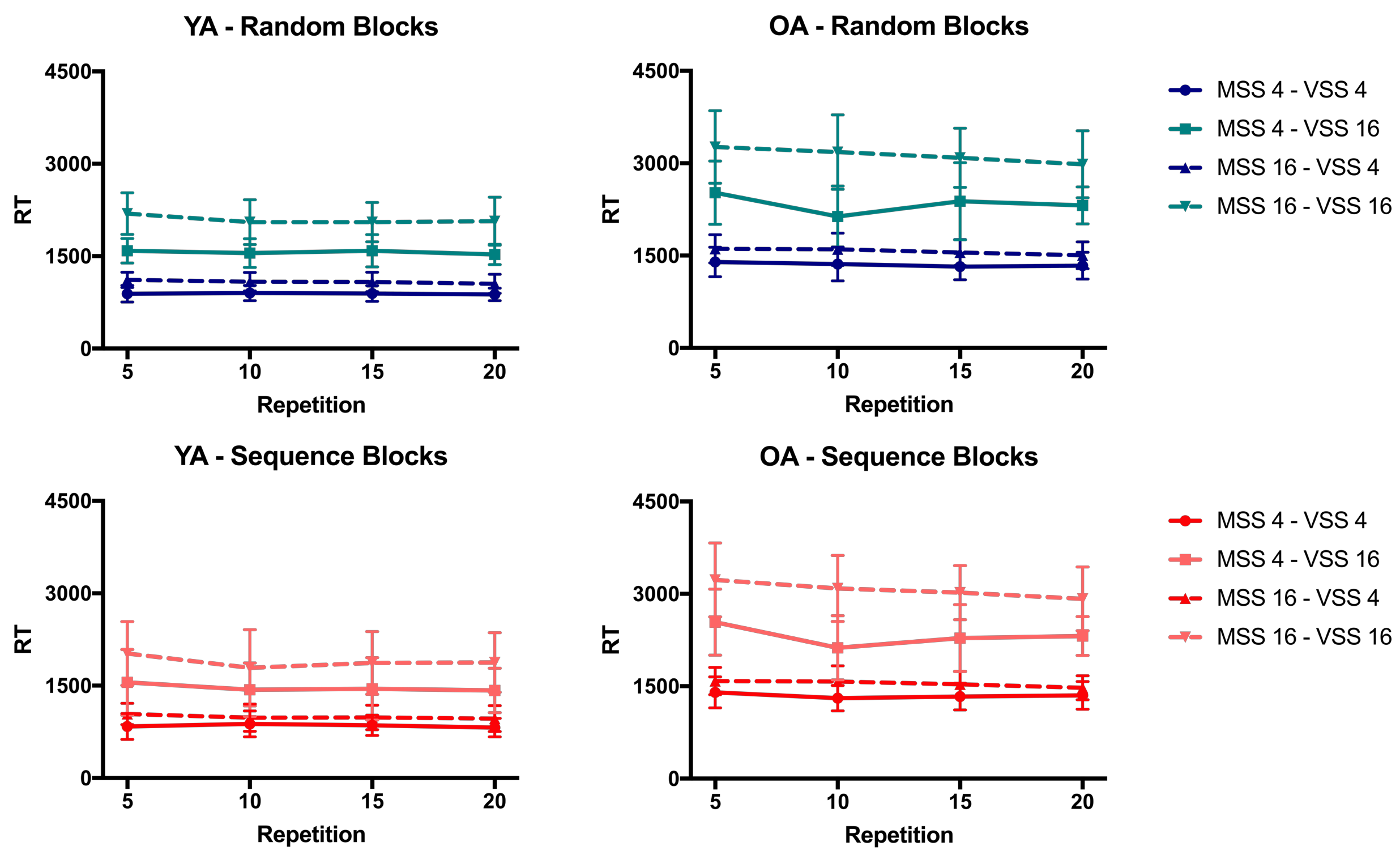


Figure S2: Mean reaction times (RT) for younger adults (YA) and older adults (OA) are plotted as a function of repetitions, comparing conditions with smaller (4) and larger (16) visual set sizes (VSS) and memory set sizes (MSS) in blocks with random (left) and repeating (right) target sequences.

Observers knew about the sequence from the beginning of Experiment 2 and the sequential order was presented first in the memorization phase prior to the search task. Presumably, explicit memory for target order associations was established fast and within a single of few exposures of the sequence. Therefore, the learning effect did not increase much further over time. Only in older adults, there is evidence for a small increase in the learning effect over trials when the target sequence was relatively long.
