# Supplementary material for: Order, please! Explicit sequence learning in hybrid search in younger and older age
Source: Mem Cognit. 2021 Apr 19;49(6):1220–35. doi: 10.3758/s13421-021-01157-2 (PMC8313466; doi:10.3758/s13421-021-01157-2)
Supplement: Supplementary file 2 — (DOCX 21 kb) [file 13421_2021_1157_MOESM2_ESM.docx]

**Supplementary Material I**

**Post-Experiment Questionnaire**

After participating in Experiment 1 (incidental learning), our participants filled out a questionnaire. We asked them four questions to assess general task representation and strategy and, in particular, whether they have acquired explicit knowledge about the repeating target sequence (Q3). The questionnaire looked as follows:

*Please answer the following questions about the experiment. We are asking about your subjective experience with doing the task, so there are no right or wrong answers.*

*Q1. What do you think this experiment was about?*

*Q2. Did you find the task difficult? Did you find some parts of the experiment more difficult than others and if so, why?*

*Q3. Did you notice any repeating sequences in the order of objects you had to look for? If so, did you use this knowledge during the experiment? Can you describe one or more of the sequences or parts of the sequences?*

*Q4. Did you use any other strategies during this experiment?*
